# Ongoing reepithelialization after keratopigmentation

**DOI:** 10.5935/0004-2749.2025-0193

**Published:** 2025-11-04

**Authors:** Giovanni Garottii, Nicole Bulgarão Maricondi de Almeida, Newton Kara-Junior

**Affiliations:** 1 Ophthalmology Department, Hospital das Clinicas, Faculdade de Medicina, Universidade de São Paulo, São Paulo, Brazil

Keratopigmentation (Figure), also referred to as corneal tattooing, is a procedure involving the insertion of pigment into the corneal stroma^(1)^. It can be used to improve the cosmetic appearance of blind eyes^(2)^, thereby positively influencing patient self-esteem^(3)^.Reported complications include pigment fading, uveitis, and corneal neovascularization^(1^,^2)^.

**Figure 1:**
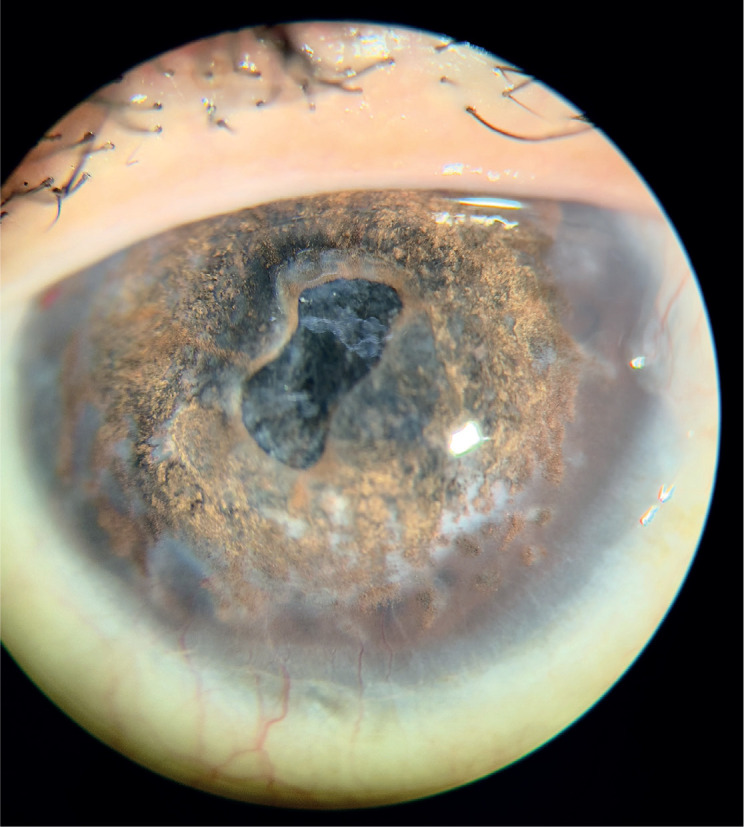
Slit lamp view of blind eye reepithelialization post Keratopigmentation procedure
